# A Survey on Knowledge, Prevention, and Occurrence of Sexually Transmitted Infections among Freshmen from Four Italian Universities

**DOI:** 10.3390/ijerph19020897

**Published:** 2022-01-14

**Authors:** Luca Cegolon, Melania Bortolotto, Saverio Bellizzi, Andrea Cegolon, Luciano Bubbico, Giuseppe Pichierri, Giuseppe Mastrangelo, Carla Xodo

**Affiliations:** 1 Occupational Medicine Unit, Department of Medical, Surgical & Health Sciences, University of Trieste, 34129 Trieste, Italy; 2 Public Health Department, University Health Agency Giuliano-Isontina (ASUGI), 34128 Trieste, Italy; 3 FISPPA Department, Padua University, 35137 Padua, Italy; melania.bortolotto@unipd.it (M.B.); carla.xodo@unipd.it (C.X.); 4 Independent Researcher, 1200 Geneva, Switzerland; saverio.bellizzi@gmail.com; 5 Department of Political, Social & International Relationships Sciences, University of Macerata, 62100 Macerata, Italy; andrea.cegolon@unimc.it; 6 Department of Sensorineural Disabilities, INAPP/Italian Institute of Social Medicine, 00198 Rome, Italy; l.bubbico@inapp.org; 7 Microbiology Department, Kingston Hospital NHS Foundation Trust, Kingston upon Thames KT2 7QB, UK; giuseppe.pichierri@nhs.net; 8 Department of Cardiac, Thoracic, Vascular Sciences & Public Health, Padua University, 35128 Padua, Italy; giuseppe.mastrangelo@unipd.it

**Keywords:** sexually transmitted infections, adolescents, young adults, university students, sexual health education, sexual health knowledge, sexual health prevention, history of sexually transmitted infections

## Abstract

**Background**. The peak of sexually transmitted infections (STI) among adolescents/young adults suggests a low level of prevention. In order to assess whether the level of sexual health education (SHE), received by several channels, was effective at improving sexual behaviors, we conducted a survey among freshmen from four Italian universities. **Methods**. This observational cross-sectional study was carried out with an anonymous self-reported paper questionnaire, administered during teaching lectures to university freshmen of the northern (Padua, Bergamo, and Milan campuses) and southern (Palermo campus) parts of the country. Knowledge of STI (a linear numerical score), knowledge of STI prevention (dichotomous variable: yes vs. no) and previous STI occurrence (polytomous variable: “no”; “don’t know”; “yes”) were the outcomes in the statistical analysis. **Results**. The final number of freshmen surveyed was 4552 (97.9% response rate). The mean age of respondents was 21.4 ± 2.2 years and most of them (70.3%) were females. A total of 60% of students were in a stable romantic relationship. Only 28% respondents knew the most effective methods to prevent STI (i.e., condom and sexual abstinence), with a slightly higher prevalence of correct answers among females (31.3%) than males (25.8%). Students with history of STIs were 5.1%; they reported referring mostly to their general practitioner (GP) (38.1%) rather than discussing the problem with their partner (13.1%). At multivariable analysis, a significantly higher level of STI knowledge was observed in older students (25+ years of age), biomedical students, and those from a non-nuclear family; lower levels were found among students of the University of Palermo, and those who completed a vocational secondary school education. Those who had less knowledge about the most effective tools to prevent STIs included males, students from the University of Palermo, students registered with educational sciences, economics/political sciences, those of foreign nationality, and those whose fathers had lower educational levels. The risk of contracting a STI was significantly lower only in students not in a stable relationship (relative risk ratio, RRR = 0.67; 95% confidence interval, 95%CI = 0.48; 0.94), whereas such risk was significantly higher in students with higher STI knowledge (RRR = 1.15; 95%CI = 1.08; 1.22). **Discussion and Conclusions**. University freshmen investigated in this study had poor knowledge of STIs and their prevention. Unexpectedly, those with higher levels of knowledge had an increased risk of STIs. There have been no educational interventions—with good quality and long-term follow-ups—that increased the confidence that such SHE programs could have population level effects. A new high-quality study is therefore recommended to assess the effectiveness of an intervention generating behavioral changes; increasing only STI knowledge may not be sufficient.

## 1. Background

Sexually transmitted infections (STIs) are considerable public health threats across all ages in high-income countries, particularly among teenagers [1]. According to the World Health Organization (WHO), their prevalence and incidence remain high, even for the four curable STIs—chlamydia, gonorrhea, trichomoniasis, and syphilis—with over one million new infections diagnosed each day worldwide on average in 2016 [2,3,4].

More than 30 different bacteria, viruses, and parasites are known to be transmitted via vaginal, anal, and oral sex, while some can be spread through blood or blood products. Many STIs, such as syphilis, HIV, hepatitis B, chlamydia, gonorrhea, and herpes, can also be transmitted from mother-to-child during pregnancy and childbirth [4]. STIs can cause an inflammation of the cervix, urethra, and vagina, as well as genital ulceration. Gonorrhea and chlamydia also cause rectal and pharyngeal infections and complications, such as pelvic inflammatory disease, chronic pelvic pain, ectopic pregnancy, and infertility. Syphilis causes neurological and cardiovascular disease in adults, stillbirth, neonatal death, premature delivery, or severe disability in babies. Chlamydia, gonorrhea, syphilis, and trichomoniasis are also associated with an increased risk of acquiring and transmitting HIV [4]. The impact of STIs on adolescents is particularly concerning, as individuals in this sub-group typically have high sexual drives and lower levels of education and sexual experience. Moreover, some STIs can have detrimental effects on the reproductive capacities of young individuals.

Despite making up approximately 25% of the sexually active population, individuals aged 15–24 account for half of the 20 million incident cases of STIs reported annually in the USA [5]. In Italy, where a sentinel surveillance system for STI relies on a network of 12 clinical centers spread across the country, the percentage of STI cases in the population aged 15–24 in 2018 was 17.9% of the total (rather than 50%, as in the USA) [6].

Modifying risk-taking behaviors is a difficult task, especially in younger ages [2]. According to recent surveys, the first sexual intercourse among Italian adolescents occurs at 15.6–16.0 years of age, on average, and frequently without protection [4]. Therefore, these young individuals should receive access to sex education before then. Schools, especially lower or higher secondary schools, are the easiest places to reach young people. Furthermore, schools are strategic settings that could be used to promote health in young people, as recommended by the WHO [7,8,9].

Sexual health education (SHE) is grounded in internationally recognized human rights and it plays a central role in the preparation of young people to achieve safe, productive, and fulfilling lives [10]. SHE is included among the competences of the European Union (EU), within a wider framework of public health, as established by article 168 of the EU treaty [11,12]. The Article 11 of the European Social Chart enforces member states to provide counselling and infrastructure for the delivery of health promotion and SHE is a fundamental element used to promote health in young people. Neglecting these public health needs by member states constitutes a violation of the European Social Chart [12]. Nonetheless, the EU does not have executive power to propose SHE policies within each individual EU member state. This has led to heterogeneity inside the EU, with SHE being mandatory in most member states (especially in northern Europe), but not in a handful of them, including Italy [12,13].

In March 2017, political pressures prompted the Department of Education of the British government to announce that sex and relationship education (SRE) would have to be officially introduced in British primary schools by September 2019 [14]. SRE enables children and young adults to gradually acquire information, competences, and values, to understand and fully enjoy their own sexuality, manage safe and satisfactory relationships, and be responsible for the health of themselves and others [15].

Notwithstanding this progress, three recent literature reviews reported evidence of a different understanding on the issue.

A comprehensive review of school-based interventions to improve the sexual-health of adolescents was published in 2017. Authors have systematically identified and synthesized evidence-based recommendations across 37 reviews, from 224 relevant trials, and quasi-experimental studies, including the highest quality and the most highly cited reviews in the field of sexual health. Methodological weaknesses—including lack of randomization, insufficient follow-up, small sample sizes, and lack of replication studies—were identified by many reviewers, along with weak and inconsistent evidence on behavioral effects. However, a clear conclusion could also be drawn. Sexual-health and relationship interventions focusing exclusively on sexual abstinence were not effective in modifying the sexual behaviors of school students [16].

An intervention review on randomized controlled trials (RCTs), including cluster RCTs, examined the effectiveness of universal school-based interventions targeting multiple risk behaviors among children and young people up to 18 years of age. Among a total of 34,680 titles identified, 27,691 articles screened, and 424 full-text articles assessed for eligibility, a total of 70 eligible studies were included in the review. The evidence was strongest for the efficacy of universal school-based interventions in preventing tobacco use, alcohol misuse, consumption of illicit drugs, antisocial behavior, and in improving physical activity among young people. In contrast, the evidence was less conclusive for the effect of health promotion interventions on risky sexual behaviors (OR = 0.83, 95%CI 0.61; 1.12; *p* = 0.22; *n* = 6 studies; 12,633 participants; I^2^ = 77%; low-quality evidence) as well as cannabis use or unhealthy diets [17].

In the third review, randomized and quasi-experimental evaluations addressing school-based interventions were examined to ascertain whether such interventions could promote young people’s sexual health. Because of inconsistent reporting, a meta-analysis was possible for only three RCTs, which provided some evidence that school environment interventions may delay the sexual debut (pooled OR = 0.5) [18].

Despite most sexual health studies targeting secondary school students, first year university students represent an important group in this context, because they are young adults, attempting to be independent from their families, usually already sexually active, and keen to new sexual experiences. Although SHE programs are no mandatory in Italian schools, students can receive SHE in many other ways. This can include, for example, direct experience, peer group, friends and sexual partners, external courses or activities, university courses, or online resources [19,20].

### Aim

The above-mentioned literature gap requires further evidence. Therefore, the main objective of this study was to explore the sexual health knowledge (SHK) of first year students from four Italian universities, investigating whether their knowledge, reflecting SHE received by different channels, was effective at preventing STIs.

## 2. Methods

### 2.1. Study Design and Questionnaire

We ran a cross-sectional observational study on freshmen at four Italian universities from the northern (Padua, Bergamo, and Milan) and southern (Palermo) part of the country. The final number of freshmen surveyed was 4552 (97.9% response rate). Subjects were recruited by convenience sampling based on individual negotiations with the respective academic staff. Students enrolled in educational sciences, foreign language/literature, biology, chemistry, medicine, natural sciences, engineering, architecture, political sciences and economics were selected to participate in this survey.

The survey was conducted in 2012 via an anonymous self-reported paper questionnaire. Since there was no validated tool, a new questionnaire (already published elsewhere [20]) was designed by experts of health education (CX and MB). The same two experts and another academic educator (AC) reviewed the survey instrument, reducing the initial number of questions to 78 and contributing to improve its quality. The questionnaire was in Italian, as alternative versions in languages other than Italian were deemed unnecessary.

To maximize the uptake and response rate, the survey was run during academic lecture times. One hour (on a single occasion) was given to the students to complete the questionnaire.

The questionnaire was not intended as a psychometric instrument with Likert-scale or Likert-type items; therefore, Cronbach tests were not performed. The tool collected plain descriptive information, such as personal data (sex, age, residence, nationality, undergraduate course of study, and previous education), family data (nationality, education and occupation of mothers and fathers, type of family, number of siblings, older sibling of same sex), and information on their current romantic relationships (being in love, length of relationship, and place of meeting). Furthermore, several multiple-choice questions were recoded as follows.

### 2.2. Knowledge on STI

Question 78 asked, “*Please indicate which of the following diseases can be transmitted by sex*”. The multiple answer options were the following: “Gonorrhea; Mononucleosis; Syphilis; AIDS; HPV (Human Papilloma virus); Hepatitis C (HCV); Hepatitis B (HBV); Hepatitis A (HAV); Genital Herpes; Streptococcal infection; Venereal lymphogranuloma; Wart; Psoriasis; Candidiasis; Tuberculosis”. The interviewees had to answer whether each of the above diseases were:

- Sexually transmittable;
- Non sexually transmittable; or
- Whether (s)he did not know.

With the exception of psoriasis (negative control), all of the above were STIs. A value of 1 was assigned each time the above diseases were considered, “*Sexually transmittable*”. In case the student answered, “*Non sexually transmittable*” or “*I do not know*”, a value of 0 was assigned to the respective disease. All values were then summed up to create a linear numerical score ranging from 0 to 15, which was used as outcome 1 in the statistical analysis.

### 2.3. Knowledge on Prevention of STI

Question 76 asked, “*Which of the following are the most efficacious methods to prevent sexually transmitted infections*?” The multiple answer options were the following: basal temperature thermometer; oil; anti-contraceptive pill; billing’s method; morning after pill; diaphragm; interrupted coitus; spermicide; condom; transparent membrane; abstinence; contraceptive plaster; male pill; ulipristal acetate; cervical cap; vaginal washing; contraceptive sponge. Interviewees could provide up to four answers, which could either be:

- Correct answers, where, “sexual abstinence” and “condom” (simultaneously) were indicated, even if additional options were ticked.
- Wrong answer: any other combination.

Knowledge of STI prevention became a dichotomous variable coding 0 (wrong answer) or 1 (correct answer), which was used as outcome 2 in the statistical analysis.

### 2.4. STI History

Students were also asked (question 69) whether they had ever contracted a STI. There were three pre-classified answers: “No” (coded as 0); “Don’t know” (code = 1); and “Yes” (code = 2). Those who answered “Yes” were then asked, “*What did you do when you contracted a STI in the past*?” Up to three different answers could be chosen on the questionnaire, among the following:

- I asked a friend for advice;
- I referred to a consultant gynecologist;
- I tried to solve it on my own;
- I went to my general practitioner (GP);
- I went to accident and emergency (A&E);
- I discussed the issue with a family member;
- I discussed the issue with my partner.

Answers to question 69, on whether students had ever contracted a STI, were coded as a three-level polytomous variable that was used as outcome 3 in the statistical analysis.

### 2.5. Statistical Analysis

Outcome 1 (STI knowledge) was employed as a dependent variable in a multiple linear regression model (model 1). Outcome 2 (STI prevention knowledge) was the dependent variable in a multiple logistic regression model (model 2). The predictors were all factors (sociodemographic profile, educational level, family background, and university campus) shown in Table 1. Results were expressed as regression coefficients (RC) for multiple linear regression (model 1) or odds ratio (OR) for multiple logistic regression (model 2), with the corresponding 95% confidence interval (95%CI). A multinomial regression model (model 3) was fit for outcome 3, where the dependent variable had three different possible outcomes; the category “*No*” was used as the baseline comparison group (a “pivot”) and the other categories were separately regressed against the pivot outcome. The regression results were displayed in terms of relative risk ratios (RRR) with the corresponding 95%CI.

In all models, backward stepwise selection of variables was used—*p* < 0.05 was the criterion—in order to reduce the confounding bias. Missing values were excluded and a complete case analysis was performed.

In order to increase their representativeness, results of the multivariable regression analyses were weighted for the distribution by age and sex of the Italian census population of 2011.

STATA 14.2 (StataCorp LLC 4905 Lakeway Drive, College Station, TX, USA) was used as the statistical software package.

### 2.6. Ethical Considerations

Approval to conduct the study was obtained from the Research Ethics Committee (REC) of the University of Padua (Prot. REC-UNIPD 7/5/2020) and the research methodology was in accordance with the relevant guidelines and regulations. Since all students were 18 years of age or older, parental consent was not required. Informed consent for study participation was obtained from the study subjects.

## 3. Results

### 3.1. Descriptive Analysis

The final number of university freshmen recruited by the survey was 4552 (97.9% response rate).

Table 1 reports the distribution of variables (sociodemographic profile, educational level, and family background by university campus) as frequencies and percentages or as mean ± standard deviation (SD). As can been seen, most survey participants were women (70.3%) and the mean age of all students was 21.4 ± 2.2 years; students of Italian nationality were 96.8% out of all respondents. A total of 21% of interviewees lived in a city center, 18.5% in a city outskirt, 17.0% in a town with more than 15,000 inhabitants, and 43.5% in a small town with less than 15,000 people.

A total of 83.7% of the respondents had a nuclear family; 18.6% reported being a single child, 53.4% had one sibling, 22.0% two siblings, and 6.1% three or more siblings. A total of 3.3% of students had a mother of foreign nationality, while 2.7% had a father of foreign nationality. Moreover, 18.0% of the students’ fathers and 16.0% of students’ mothers had a postgraduate education. A total of 34.4% of fathers had educational levels limited to lower secondary schools, while the respective proportion for mothers was 34.6%.

A total of 59.5% of interviewees received classical/scientific secondary school education or already had a university degree, whereas 13.7% had a secondary education in art, foreign languages, or socio-pedagogical classes, and 26.8% came from a vocational secondary school. The distribution by university was as follows: University of Padua 40.5%; University of Palermo 38.3%; University of Bergamo 14.7%; and the University of Milan 6.6%). Among university campuses, the distribution of students by undergraduate course of study was fairly homogeneous. However, no students of the course of literature/foreign language were recruited from the University of Padua. By contrast, students from the University of Bergamo were mainly enrolled in literature/foreign language courses (data not shown).

There were 60% of students in stable relationships—65.8% (=2088/3174) among females and 46.4% (=618/1333) among males. The percentage of stable relationships increased with the age of the interviewees: 56.9% among students 18–20 years old; 61.6% among those 21–24 years old; 68.9% in students 25 or older (data not shown). Among those in a romantic relationship, 90% described themselves as being in love with their respective partners, 9% did not know, and only 2% described themselves as not being in love with their partners. Stable relationships were ongoing for at least 24 months in 52% of cases, 13–24 months in 21% of cases, 7–12 months in 12% of cases, and 3–6 months in 81% of cases.

Table 2 shows the answers to the question: “*Which of the following diseases can be transmitted by sex*?” To identify areas that could benefit increasing SHE, STIs were arranged in Table 2 in descending order of “*I do not know*” (DK). More than half of the students (males as well as females) did not know that streptococcus, lymphogranuloma venereum, and hepatitis A belong to STIs. Among males, a percentage >50% did not know that candida and HPV are STIs. On the other hand, 97.2% of respondents recognized AIDS as a STI. A total of 84% of students were aware of the sexual transmissibility of herpes genitalis, with rather consistent distributions by sex and the university campus. A total of 80% of respondents recognized syphilis as a STI, although with a lower proportion (70%) among students from the University of Palermo. Knowledge of HBV and HCV as STIs displayed a distribution fairly consistent across different universities. Gonorrhea was indicated as a STI by 45% of students and lymphogranuloma venereum (chlamydia) by 32.1%, with alike distribution by university and sex.

Table 3 displays the answers to the question “*Which of the following are the most efficacious methods to prevent sexually transmitted infections?*” It can be noted that, as many as 86% of the respondents (males 85% vs. females 87%) knew about the use of condoms in preventing STI. Sexual abstinence was reported as the second most effective option to prevent STIs after condoms, with a fairly consistent distribution across different university campuses and by sex of respondents.

As can be seen from Table 4, students with a history of STIs mainly referred to their GPs (38.1%). Further reference figures included a family member (15.1%), their partner (13.1%), a consultant gynecologist (13.1%), or an experienced friend (12.5%) (Table 4).

Table 5 shows the parameters of outcome 1 (mean ± standard deviation) and the distribution of outcome 2 (number and percentage of wrong and correct answers) and outcome 3 (number and percentage of the three answers to the question on whether students had ever contracted a STI) in the entire sample and in subgroups broken down by sex and university campus. The overall average score for outcome 1 was 5.3 ± 2.9 (the median was 6 and IQR: 3–7), slightly higher among female students and lower for the university of Palermo (4.8 ± 3.2) compared to the other three campuses of northern Italy. As can be seen for outcome 2, only 28% of respondents knew the most effective methods to prevent STIs (i.e., sexual abstinence and condom). The percentage of correct answers on the knowledge of STI prevention was slightly higher among females (31%) than males (28%). Concerning outcome 3, it can be noted (column under the heading “Yes”) that STIs were more frequent among female students and in those from Padua University. All other campuses in northern and southern Italy showed fewer frequencies of STIs.

### 3.2. Outcome Analysis

Table 6 presents, in two columns, the outcomes (1 = STI knowledge; 2 = knowledge of STI prevention), in the rows—the predictors, and at the interceptions of rows and columns—the results of multivariable regression analyses. Several variables not appearing in the rows were removed by the stepwise backward selection. The results were the RCs (with 95% confidence interval, 95%CI) in the first column and ORs (with 95%CI) in the second column.

Few variables had an effect on both outcomes. An example was “*Undergraduate course of study*”. Always using the group of biology/medicine/chemistry/natural sciences as reference categories, all other undergraduate courses showed significantly lower knowledge on outcomes 1 and 2; in particular, freshmen in educational sciences showing the lowest knowledge of STI (RC = −0.79; 95%CI: −1.07; −0.52) and STI prevention (OR = 0.57; 95%CI: 0.45; 0.71). The second example was “*university campus*”. Using as reference the University of Padua, freshmen at the University of Palermo were found to have significantly less knowledge of both STI (RC = −0.89; 95%CI: −1.12; −0.67) and STI prevention (OR = 0.43; 95%CI: 0.35; 0.51), but, again, they were not at risk of STI.

The remaining variables had one-to-one effects. Knowledge of STI was higher in students aged 25+ years (RC = 0.61; 5%CI: 0.27; 0.95) compared with those aged <21 years, and in those from a non-nuclear type of family (RC = 0.55; 0.31; 0.80); whereas it was lower among students who completed a vocational secondary school education (RC = −0.35; 95%CI: −0.57; −0.14). Knowledge on the most efficacious preventative tools against STIs was significantly lower in males (OR = 0.66; 95%CI: 0.54; 0.81), in students of foreign nationality (OR = 0.19; 95%CI: 0.09; 0.41), in those whose fathers had their education limited to secondary school (OR = 0.64; 95%CI: 0.50; 0.82) or junior secondary school (OR = 0.69; 95%CI: 0.53; 0.90), or who were employed as technicians (OR = 0.74; 95%CI: 0.55; 0.99).

Table 7 displays, in two columns, the results of the multinomial regression analysis (model 3), investigating the risk factors associated with a history of STIs. Both group of students either aware (second column heading = “*Yes*”) or not fully aware (first column heading = “*Don’t know”*) of having contracted a STI in the past were compared with the same base “*No*” (i.e., students confident not to have contracted STI). A different epidemiologic pattern was found. In the first column of Table 7, including students not confident of having contracted a STI in the past, the risk of STI increased among freshmen of the University of Palermo (RRR = 1.80; 95%CI: 1.08; 3.03); and decreased in those not in a stable romantic relationship (RRR = 0.56; 95%CI: 0.34; 0.94). In the second column, under the heading “*Yes*”, a value of RRR < 1 (indicating a negative association, i.e., a preventive effect) was observed in male students (RRR = 0.62; 95%CI: 0.43; 0.89), in those whose fathers were under 50 years of age (RRR = 0.45; 0.28: 0.73), and among interviewees not in a relationship (RRR = 0.67; 95%CI: 0.48; 0.94), while the risk of contracting a STI increased with higher level of outcome 1 (knowledge of STI; RRR = 1.15; 95%CI: 1.08; 1.22). The latter variable was added as a further predictor in model 3 of the multinomial regression. Knowledge of STI prevention (outcome 2) was also introduced as a predictor in model 3, but the variable was removed by the backward stepwise selection.

## 4. Discussion

### 4.1. Key Findings

The present survey evidenced that:

- The overall knowledge of STIs among sample students was low (Table 2);
- Students with histories of STIs referred mostly to their GPs (38.1%), followed by a consultant gynecologist (13.1%), a family member (15.1%), or an experienced friend (12.5%), whereas discussing the problem with their partner was reported only by 13.1% freshmen (Table 4);
- Only 28% of the students knew the most effective prophylactic device to prevent a STI (i.e., sexual abstinence and condom); the percentage being slightly higher for females (31.3%) than males (25.8%) (Table 5);
- Significantly lower levels of STI knowledge were found among males, students at the University of Palermo, those who completed a vocational secondary school education, and non-Italian nationals (Table 6);
- Categories of individuals significantly less knowledgeable on the most effective options in preventing STI included males, students at the university of Palermo, students in educational sciences, students in economics/political sciences, those of foreign nationality, those with two siblings, and those whose fathers had lower educational levels (Table 6);
- The risk of contracting a STI was found to be lower in males, in students with fathers aged < 50 years and in students not in a stable relationship; by contrast it increased with higher knowledge of STIs (Table 7).

### 4.2. Comparison of Findings with the Relevant Literature

#### 4.2.1. STIs Knowledge

While HIV and syphilis were correctly recognized as STIs by 97% and 80% respondents in the present survey, gonorrhea was correctly identified by 44.9% of them and Chlamydia (lymphogranuloma venereum) by 32.1%, with rather similar distribution by university campus and sex of respondents. Genital warts, correctly recognized as STI by 15.0% of interviewees, was by far the most unknown STIs in our survey.

A systematic review on young European adolescents aged 13–20 years reported a relatively higher knowledge of HIV/AIDS that has been probably influenced by the extensive preventative and informative campaigns deployed at international level over the last 30 years [21,22,23,24]. Aside HIV/AIDS, a low level of knowledge of STIs, their diffusion, means of contagion, preventative methods, risky behaviors, and health impact was reported across Europe [25]. However, in Britain individuals aged 15 to 24 years reportedly experienced the highest rates of chlamydia, genital herpes, and genital warts, whereas the highest incidence rates of gonorrhea and syphilis among men affected older age groups [26]. Among heterosexuals diagnosed with STIs in 2015 in the UK—62% (62,191/100,165) with chlamydia, 52% (9088/17,414) with gonorrhea, 51% (32,113/62,547) with genital warts and 41% (12,591/30,658) with genital herpes—were individuals in this age group. Although the overall number of STI diagnoses in those aged 15 to 24 years have risen considerably in the last five years, there has been a decline recently in cases of genital warts in young females associated with Human Papillomavirus vaccination [26]. Chlamydia infection is the most common STI across Europe, featured by an incidence rate of 146 per 100,000 in 2018 across the entire EU [27,28]. The geographical distribution of Chlamydia varies importantly though, with notifications being considerably higher among Northern European countries, especially in the UK, which accounted for 60% of all cases notified in 2018 in the entire EU [28]. As a result of this, whilst 20 EU countries adopted a comprehensive surveillance system (with mandatory notifications) for chlamydia, 6 countries—including Italy—employed a sentinel surveillance system [27].

If “*I don’t know*” (DKs) were added to negative answers, TB becomes the least disease associated with sex in the present survey. TB is not spread through sexual intercourse or kissing or other touch. TB bacteria are spread through the air when a person with TB coughs, sneezes, speaks, or sings. Several associations between the risk for TB and lifestyle factors have been identified. For example, unmarried persons are at higher risk of TB than married individuals. Moreover, HIV co-infection is the strongest known risk factor for disease progression from latent to active TB [29].

The present study evidenced relevant cross-regional differences within Italy on SHK in terms of STIs prevention. In particular, students of the university of Palermo consistently showed significant lower levels of SHK, somehow confirming the cultural barriers for the implementation of SHE in Southern Italy [30], as already suggested by a survey on 2867 secondary schools’ students from the city of Genoa and Lecce [31]. Sexuality is indeed still considered a taboo in Italy and discussing it in the family is difficult, especially in the southern part of the country [30,32]. This was somehow endorsed also in the present study, where female students of the University of Palermo were remarkably less inclined than their colleagues from Northern Italy to refer to a family member or their partner in case of STI.

#### 4.2.2. STI Prevention Knowledge

Although condom was considered by far (86.2%) the most effective option against STIs in the present survey, with similar distribution by university campus and sex of respondents, only 28.0% of the students acknowledged both condoms as well as sexual abstinence as the most efficacious prophylactic tools to prevent STIs. Likewise, only 22% of 2867 students from the cities of Lecce and Genoa knew that both condoms and sexual abstinence were the most reliable methods to prevent STIs [31].

In the study here presented, the contraceptive pill was the third most considered preventive option rated effective to prevent STIs; this was also confirmed by a survey amongst undergraduate biomedical students of Palermo [21]. In the latter study however, 7% students did not use any specific contraception and a large percentage reported using contraceptive pills and vaginal rings, certainly effective to prevent unintended pregnancies but ineffective against STIs [21]. In a survey from the Emilia Romagna region, a considerable proportion of students was convinced that contraceptive pills (22.1% vs. 23.5%), spermicides (14.6% vs. 46.9%), intra-uterine device (IUD) (18.25 vs. 39.1%), coitus interruptus (8.1% vs. 38.1%) and morning after pill (9.8% vs. 30.0%) were respectively “*partly safe*” versus “*very safe*” to prevent STIs [33]. Likewise, in another survey on 139 students attending the 3rd class of a secondary school in Milan (Lombardy Region, Northern Italy), albeit condom was reported by 92.1% respondents as a reliable method to prevent STIs, the contraceptive pill (24.5%), morning-after pill (20.1%), diaphragm (11.5%) and coitus interruptus (10.8%) were also wrongly indicated, casting legitimate concerns on the likely conceptual confusion between contraception and STIs prevention among an important part of Italian adolescents [34].

In this study, only 13.1% of students who contracted a STI discussed the problem with their partners (Table 4). However, partner notification can reduce infection spread according to Public Health England [26].

In the present study, biomedical students had a significantly higher knowledge and understanding of both STIs and the most effective ways to prevent them, a finding consistent with the existing literature [8,21,35,36,37]. In a study involving 2074 students from the University of Madrid, medical students were less sexually active, had their sexual debut at a later stage, and had less sexual partners as compared to law students [36]. Biomedical students were considered for peer education in schools, to maximize the delivery of SHE [1].

#### 4.2.3. STI History

The cross-sectional design, without a time dimension in the present study, could not explain the paradoxical result of the increasing risk of STIs with higher STI knowledge. Although it is well known that gaps exist between knowledge of STIs and good preventive sexual practices, it seems more likely that SHK was primarily gained by direct experience of STIs, rather than from acquiring knowledge to prevent STI infection.

Only 13% of students with history of STIs reported discussing the problem with their partners, thereby increasing the risk of spreading the infection. The lower risk of past STIs among males indicated that, in the present study, STIs were transmitted mainly by heterosexual contact or lesbian sex. The key to preventing the spread of STIs, especially in epidemics sustained mainly by heterosexual transmission, is modifying sexual behaviors. Multiple (especially concurrent) sexual partnerships, combined with unprotected sex, is a critical risk factor for the spread of STIs [38].

About 40% of our students were not in a stable relationship, and sexual abstinence is a key factor to contain the risk of STIs, since young people aged 16–24 years are more likely to report a new or two or even more sexual partners of the opposite sex in the past year [26].

The so called ‘ABC’ approach, where “A” stands for abstinence or delay of sexual activity, “B” for be faithful to one partner, and “C” for condom use, is receiving growing interest [38]. Although “*be faithful*” implies monogamy, it also includes reductions in casual and multiple sexual partnerships. Behavior changes to prevent STIs have mainly promoted condom use or abstinence, whereas the containment of the number of sexual partners remains a neglected component of ABC [38]. Albeit, prospective studies have shown that condoms can reduce the STI risk by about 80–90%, in real life, they are often used incorrectly or inconsistently [39,40]. Likewise, although abstinence may be a viable option for many young people, for others, it may be an unrealistic expectation. According to a recent review of reviews [16], interventions promoting abstinence-only were effective at improving knowledge about STIs, how sexual abstinence can protect against STIs, and the health risks and consequences of unprotected sex, including unintended pregnancy, but were not effective at modifying risky sexual behavior. Furthermore, there was even some evidence that such programs may increase sexual activity, the rates of STIs, and unintended pregnancies [16].

### 4.3. Sexual Health Education

Recent surveys reported that the first sexual intercourse experiences occurred at the average age of 15.6–16.0 years among Italian adolescents, frequently without protection [4]. For this reason, these young individuals should be able to access sex education before that age. Schools, lower or upper secondary schools, are the easiest places to reach young people. Similar to therapeutic treatments, which are approved by regulatory agencies following successful RCTs, preventative interventions should also prove to be effective in a similar fashion. No educational interventions, of good quality and long-term follow-ups, would increase the confidence that such programs could have population level effects. A new, high-quality study is therefore recommended, with objective measures of health-related outcomes. This study should include a detailed description of the intervention components, and chlamydia infection (the most prevalent STI across Europe) could be used as a proxy sentinel event to test the efficacy of the sexual health program, screening the urine of study participants before and after receiving the educational intervention.

### 4.4. Sexual Health Clinics

School-based/linked sexual-health services are “*services or clinics provided in schools; services located near schools that conduct outreach work within those schools; or services located near schools that liaise formally with those schools. The interventions of interest are those delivered to individuals who attend the services on a voluntary basis, and do not include either classroom interventions*” [41]. According to the Committee for the Rights of the Child (CRC) all adolescents “*should have access to free, confidential, adolescent-responsive, and non-discriminatory sexual and reproductive health services, information and education*” [42]. Most EU member states offer free access to community reproductive and sexual health services, considered critical for adolescents (especially girls) [42,43]. For instance, youth centers (free of charge for individuals under 25 years of age) have been implemented in Estonia since 1991–1992, delivering screening and treatment for STIs, counselling on contraception and STI prevention as well as support to teachers for school SHE programs [44].

The creation of sexual health clinics implies instituting specialist training programs for sexual health professionals who should cooperate with families, school teachers, local pharmacies and other allied health professionals (public health specialists, GPs, consultant gynecologists, health educators) to promote sexual health within an integrated multi-agency framework [43].

## 5. Conclusions

The level of SHK among first year students at four Italian universities was ineffective at preventing STIs. Therefore, a new high-quality study is warranted to assess the effectiveness of preventive sexual health interventions, promoting behavioral changes among adolescents and young adults, since only improving their SHK may not be sufficient.

## Figures and Tables

**Table 1:** Distribution of sociodemographic profile, educational level, and family background. Number and column percentage (%) or mean ± standard deviation.

Variables	Classes	Number (%)
**Sex** (Missing: 8)	Female	3194 (70.3)
	Male	1350 (29.7)
**Age (years)** (Missing: 24)	Mean	21.4 ± 2.2
	<21	2125 (46.9)
	21–24	1956 (43.2)
	25+	447 (9.9)
Nationality	Italian	4405 (96.8)
	Non-Italian	147 (3.2)
**Undergraduate** **course of study**	Educational sciences	1591 (35.0)
	Literature/foreign language	516 (11.3)
	Biology/medicine/chemistry/natural sciences	817 (18.0)
	Engineering/architecture	971 (21.3)
	Political sciences/economics	657 (14.4)
**Previous education** (Missing: 84)	Scientific/classic/university degree	2659 (59.5)
	Language/socio-pedagogical/artistic	612 (13.7)
	Vocational (technical)	1197 (26.8)
**Residence** (Missing: 29)	City center	951 (21.0)
	City Outskirt	838 (18.5)
	Town > 15,000 inhabitants	768 (17.0)
	Town < 15,000 inhabitants	1966 (43.5)
**Father’s age (years)** (Missing: 587)	<50	795 (20.1)
	50–54	1384 (34.9)
	55–59	1090 (27.5)
	60+	696 (17.6)
**Mother’s age (years)** (Missing: 552)	<45	386 (9.7)
	45–49	1294 (32.4)
	50–54	1345 (33.6)
	55+	975 (24.4)
**Mother’s nationality** (Missing: 251)	Italian	4158 (96.7)
	Non-Italian	143 (3.3)
**Father’s nationality** (Missing: 273)	Italian	4163 (97.3)
	Non-Italian	116 (2.7)
**Father’s education** (Missing: 148)	University/more	791 (18.0)
	Secondary	2097 (47.6)
	Lower	1516 (34.4)
**Mother’s education** (Missing: 104)	University/more	711 (16.0)
	Secondary	2200 (49.5)
	Lower	1537 (34.6)
**Father’s Occupation** (Missing: 361)	Manager/professionals	828 (19.8)
	Technical employees	2077 (49.6)
	Generic employees	657 (15.7)
	Other	629 (15.0)
**Mother’s occupation** (Missing: 306)	Manager/professionals	747 (17.6)
	Technical employees	1455 (34.3)
	Generic employees	1837 (43.3)
	Other	207 (4.9)
**Type of family** (Missing: 17)	Nuclear	3796 (83.7)
	Other	739 (16.3)
**Number** **of siblings**	0	846 (18.6)
	1	2429 (53.4)
	2	999 (22.0)
	3+	278 (6.1)
**Older sibling of same sex** (Missing: 4)	Singleton	846 (18.6)
	Female with at least on older sister	309 (6.8)
	Male with at least on older brother	812 (17.8)
	Other	2581 (56.8)
**Current relationship?** (Missing: 37)	Yes	2710 (60.0)
	No	1805 (40.0)
**Have you ever contracted a sexually transmitted disease?** (Missing: 134)	No	4055 (91.8)
	Do not Know	137 (3.1)
	Yes	226 (5.1)
**Are you in love with your partner?** (Missing: 42)	Yes	2378 (89.1)
	No	64 (2.4)
	Do not Know	226 (8.5)
**Duration of the current relationship** **(months)** (Missing: 29)	<3	199 (7.4)
	3–6	218 (8.1)
	7–12	312 (11.6)
	13–24	558 (20.8)
	24+	1394 (52.0)
**Place where you first** **met your partner** (Missing: 1)	School	557 (10.6)
	Club	555 (20.5)
	Gym	87 (3.2)
	Friend’s place	664 (24.5)
	Internet	162 (6.0)
	Charity	113 (4.2)
	Other	571 (21.1)

**Table 2:** Answers to the question: “Which of the following diseases can be transmitted by sex?” Number and row percentage (%). DK = I do not know.

Diseases	Answers		
	Yes	No	DK
Warts	575 (15.0)	309 (8.1)	2943 (76.9)
Streptococcus	747 (19.6)	870 (22.8)	2199 (57.6)
Lymphogranuloma venereum	1247 (32.1)	444 (11.4)	2194 (56.5)
Hepatitis A	733 (19.1)	1051 (27.4)	2050 (53.5)
Gonorrhea	1737 (44.9)	339 (8.8)	1793 (46.3)
Hepatitis B	1260 (31.5)	955 (25.1)	1650 (43.4)
HPV	1954 (50.5)	337 (8.7)	1578 (40.8)
TB	327 (8.5)	2018 (52.2)	1524 (40.0)
Hepatitis C	1629 (42.2)	862 (22.4)	1366 (35.4)
Candida	2258 (58.0)	277 (7.0)	1385 (35.0)
Mononucleosis	1139 (29.3)	1791 (46.2)	948 (24.5)
Syphilis	3195 (80.0)	147 (3.7)	62 (16.3)
Genital Herpes	3369 (84.0)	157 (3.9)	491 (12.2)
HIV	4056 (97.2)	48 (1.2)	68 (1.6)
Negative control: psoriasis	305 (7.9)	1389 (36.1)	2154 (56.0)

**Table 3:** Answers to the question: “*Which of the following are the most efficacious methods to prevent sexually transmitted infections?*” (correct answers green marked). Number and percentage (%) of total subjects.

Preventative Methods	Total	Males	Females
Condom	3712 (86.4)	1084 (85.3)	2623 (86.9)
Sexual abstinence	1467 (32.2)	392 (29.0)	1074 (33.6)
Anti-contraceptive pill	1152 (25.3)	362 (26.8)	787 (24.6)
Coil	432 (9.5)	118 (8.7)	314 (9.8)
Morning after pill	367 (8.1)	174 (12.9)	190 (6.0)
Diaphragm	254 (5.6)	80 (5.9)	172 (5.4)
Contraceptive plaster	121 (2.7)	26 (1.9)	95 (3.0)
Transparent membrane	116 (2.6)	41 (3.0)	75 (2.4)
Interrupted coitus	112 (2.5)	74 (2.8)	38 (2.3)
Cervical cap	99 (2.2)	22 (1.6)	77 (2.4)
Vaginal washing	95 (2.1)	32 (2.4)	63 (2.0)
Spermicide	90 (2.0)	36 (2.7)	52 (1.6)
Basal temperature thermometer	73 (1.6)	24 (1.8)	49 (1.5)
Billing method	61 (1.3)	24 (1.8)	37 (1.2)
Male pill	12 (0.3)	7 (0.5)	5 (0.2)
Ulipristal acetate	4 (0.1)	3 (0.2)	1 (0.0)
Contraceptive sponge	17 (0.4)	9 (0.7)	8 (0.3)

**Table 4:** Answer to the question: “*What have you done when you contracted a sexually transmitted infection in the past?*” Number and column percentage by sex.

Answers	Total *	Males *	Females *
I asked advice to an expert friend	39 (12.5)	7 (11.7)	32 (12.5)
I referred to a gynecologist	41 (13.1)	1 (1.7)	40 (15.7)
I tried to resolve it on my own	15 (4.8)	7 (11.7)	8 (3.1)
I went to my GP	119 (38.1)	25 (41.7)	93 (36.5)
I went to A&E	10 (3.2)	2 (3.3)	8 (3.1)
I spoke with somebody of my family	47 (15.1)	8 (13.3)	39 (15.3)
I have spoken with my partner	45 (13.1)	10 (16.7)	35 (13.7)
**Total answers provided**	**312**	**60**	**255**

* Respondents were allowed to select multiple options; hence, the total number of answers provided (N = 312) exceeds the number of students with a history of STIs (N = 226).

**Table 5:** Outcome 1 (mean ± standard deviation, SD), outcome 2 (number and row percentage of wrong and correct answer), and outcome 3 (number and row percentage of the three answers to the question on whether students had ever contracted a STI) in the whole population and, separately, by sex and university campus.

	Outcome 1 (Range: 0–15)	Outcome 2 ^@^		Outcome 3 ^@^		
	Mean ± SD	Wrong Answer	Correct Answer	No	Don’t Know	Yes
**Total**	5.3 ± 2.9	3023 (70.4)	1273 (29.6)	4055 (91.8)	137 (3.1)	226 (5.1)
**Females**	5.4 ± 2.9	2074 (68.7)	944 (31.3)	2827 (69.8)	94 (68.6)	182 (80.9)
**Males**	5.1 ± 3.1	943 (74.2)	328 (25.8)	1222 (30.2)	43 (31.4)	43 (19.1)
**Padua**	5.8 ± 2.6	1158 (64.9)	626 (35.1)	1687 (41.6)	37 (27.0)	96 (42.5)
**Palermo**	4.8 ± 3.2	1248 (78.0)	352 (22.0)	1496 (36.9)	79 (57.7)	78 (34.5)
**Bergamo**	5.6 ± 2.7	412 (65.8)	214 (34.2)	599 (14.8)	17 (12.4)	34 (15.0)
**Milan**	5.1 ± 2.9	205 (71.7)	81 (28.3)	273 (6.7)	4 (2.9)	18 (8.0)

^@^ = 256 missing values (blanks).

**Table 6:** Results of multiple linear regression (outcome 1) and multiple logistic regression (outcome 2): regression coefficient (RC) and odds ratio (OR) with 95%CI.

Factors	Classes	Outcome 1	Outcome 2
		Linear Regression RC (95%CI)	Logistic Regression OR (95%CI)
**Sex**	Female		Reference
	Males		0.66 (0.54; 0.81)
**Age** (years)	<21	Reference	
	25+	0.61 (0.27; 0.95)	
**University** **campus**	Padua	Reference	Reference
	Palermo	−0.89 (−1.12; −0.67)	0.43 (0.35; 0.51)
**Previous** **education**	Classic/scientific/university degree	Reference	
	Vocational	−0.35 (−0.57; −0.14)	
**Undergraduate** **course of study**	Educational sciences	−0.79 (−1.07; −0.52)	0.57 (0.45; 0.71)
	Literature/Foreign language	−0.66 (−1.03; −0.29)	0.73 (0.53; 0.99)
	Biology/medicine/chemistry/natural sciences	Reference	Reference
	Engineering/architecture	−0.68 (−0.96; −0.41)	0.79 (0.62; 1.00)
	Political science/economics	−0.35 (−0.66; −0.04)	0.62 (0.48; 0.80)
**Nationality**	Italian		Reference
	Non-Italian		0.19 (0.09; 0.41)
**Type of Family**	Nuclear	Reference	
	Non-nuclear	0.55 (0.31; 0.80)	
**Father’s** **education**	University/more		Reference
	Secondary		0.64 (0.50; 0.82)
	Lower		0.69 (0.53; 0.90)
**Father’s** **occupation**	Manager/professionals		Reference
	Technical employees		0.74 (0.55; 0.99)

**Table 7:** Outcome 3 (three answers to the question on whether students had ever contracted a STI). Multivariable analysis of multinomial regression: relative risk ratios (RRR) with 95% confidence interval (95%CI) for answers “Don’t know” and “Yes” compared to the base outcome “No”.

Factors	Classes	RRR and 95%CI for the Answers:	
		Don’t Know	Yes
**Sex**	Female		Reference
	Males		0.62 (0.43; 0.89)
**University** **campus**	Padua	Reference	
	Palermo	1.80 (1.07; 3.03)	
**Father’s** **age (years)**	60+		Reference
	<50		0.45 (0.28; 0.73)
**Current relationship**	Yes	Reference	Reference
	No	0.56 (0.34; 0.94)	0.67 (0.48; 0.94)
**STI knowledge**	Linear numerical score		1.15 (1.08; 1.22)

## Data Availability

The datasets generated and analyzed during the current study are not publicly available, since they were purposively collected by the authors for the present study, but may be available from the corresponding author on reasonable request.

## Abbreviations

A&E	Accident and Emergency Service
EU	European Union
GP	general practitioner
HIV	human immunodeficiency virus
HAV	hepatitis A virus
HBV	hepatitis B virus
HCV	hepatitis C virus
HPV	human papilloma virus
IUD	intra-uterine device
SHE	sexual health education
SHK	sexual health knowledge
STI	sexually transmitted infection
WHO	World Health Organization

## References

[1] Duh E., Medina S.P., Coppersmith N., Adjei N., Roberts M.B., Magee M. (2017). Sex Ed by Brown Med: A Student-Run Curriculum and Its Impact on Sexual Health Knowledge. Fam. Med..

[2] Panatto D., Amicizia D., Trucchi C., Casabona F., Lai P.L., Bonanni P., Boccalini S., Bechini A., Tiscione E., Zotti C.M. (2012). Sexual behaviour and risk factors for the acquisition of human papillomavirus infections in young people in Italy: Suggestions for future vaccination policies. BMC Public Health.

[3] World Health Organization (2018). Report on Global Sexually Transmitted Infection Surveillance.

[4] World Health Organization Sexually Transmitted Infections (STI). http://www.who.int/topics/sexually_transmitted_infections/en/.

[5] Centre for Disease Control and Prevention (CDC) Adolescents and Young Adults. https://www.cdc.gov/std/life-stages-populations/adolescents-youngadults.htm.

[6] Istituto Superiore di Sanità (Notiziario) Le Infezioni Sessualmente Trasmesse: Aggiornamento Dei Dati Dei Due Sistemi Di Sorveglianza Sentinella Attivi in Italia Al 31 Dicembre 2018. https://www.epicentro.iss.it/ist/pdf/notiziario-dati-2018.pdf.

[7] WHO Regional Office for Europe and BZgA (2010) (2010). Standards for Sexuality Education in Europe—A Framework for Policy Makers, Educational and Health Authorities and Specialists. https://www.icmec.org/wp-content/uploads/2016/06/WHOStandards-for-Sexuality-Ed-in-Europe.pdf.

[8] Visalli G., Cosenza B., Mazzù F., Bertuccio M.P., Spataro P., Pel-licanò G.F., DIPietro A., Picerno I., Facciolà A. (2019). Knowledge of sexually transmitted infections and risky behaviours: A survey among high school and university students. J. Prev. Med. Hyg..

[9] Barnekow V., Buijs G., Clift S., Jensen B.B., Paulus P., Rivett D., Young I. (2006). Health-Promoting Schools: A Resource for Developing Indicators. https://apps.who.int/iris/bitstream/handle/10665/107805/E89735.pdf;jsessionid=4BC9106BFE9526781F340DF3BD8BAFEA?sequence=1.

[10] UNESCO (2018). International Technical Guidance on Sexuality Education. An Evidence-Informed Approach. https://www.who.int/reproductivehealth/publications/adolescence/comprehensive-sexuality-education/en/.

[11] Treaty L. Consolidated version of the Treaty on the Functioning of the European Union, Title XIV ‘Public Health’, Article 168. http://eurlex.europa.eu/LexUriServ/LexUriServ.do?uri=OJ:C:2010:083:0047:0200:en:PDF.

[12] European Parliament—Directorate General for European Policies (2013). Policies for Sexuality Education in the European Union. https://op.europa.eu/en/publication-detail/-/publication/17522867-38aa-4ec5-b5b4-711d96f7b900.

[13] Kundisova L., Nante N., Lorenzini C., Valeri V., Messina G., Alaimo L. (2019). Italian adolescents and sexual health: What has changed during the last 20 years?. Eur. J. Public Health.

[14] UK Department of Education Statutory Guidance Relationships Education (Primary). https://www.gov.uk/government/publications/relationships-education-relationships-and-sex-education-rse-and-health-education/relationships-education-primary.

[15] IPPF, BZgA (2018). Sexuality Education in Europe and Central Asia. State of the Art and Recent Developments. An Overview of 25 Countries.

[16] Denford S., Abraham C., Campbell R., Busse H. (2017). A comprehensive review of reviews of school-based interventions to improve sexual-health. Health Psychol. Rev..

[17] MacArthur G., Caldwell D.M., Redmore J., Watkins S.H., Kipping R., White J., Chittleborough C., Langford R., Er V., Lingam R. (2018). Individual-, family-, and school-level interventions targeting multiple risk behaviours in young people. Cochrane Database Syst. Rev..

[18] Peterson A.J., Donze M., Allen E., Bonell C. (2019). Effects of Interventions Addressing School Environments or Educational Assets on Adolescent Sexual Health: Systematic Review and Meta-analysis. Perspect. Sex. Reprod. Health.

[19] Henry J., Kaiser Family Foundation (2003). National Survey of Adolescents and Young Adults: Sexual Health Knowledge, Attitudes and Experiences. https://www.kff.org/wp-content/uploads/2013/01/national-survey-of-adolescents-and-young-adults.pdf..

[20] Cegolon L., Bortolotto M., Bellizzi S., Mastrangelo G., Cegolon A., Xodo C. (2020). Birth Control Knowledge among Freshmen of Four Italian Universities. Sci. Rep..

[21] Santangelo O.E., Provenzano S., Firenze A. (2018). Knowledge of sexually transmitted infections and sex-at-risk among Italian students of health professions. Data from a one-month survey. Ann. Ist. Super. Sanità.

[22] Grad A.I., Senilă S.C., Cosgarea R., Tataru A.D., Vesa S.C., Vica M.L., Matei H.V., Ungureanu L. (2018). Sexual Behaviors, Attitudes, and Knowledge about Sexually Transmitted Infections: A Cross-sectional Study in Romania. Acta Derm. Croat..

[23] Glasier A., Gülmezoglu A.M. (2006). Putting sexual and reproductive health on the agenda. Lancet.

[24] Shapiro J., Radecki S., Charchian A.S., Josephson V. (1999). Sexual behavior and AIDS related knowledge among community college students in Orange County, California. J. Commun. Health.

[25] Samkange-Zeeb F.N., Spallek L., Zeeb H. (2011). Awareness and knowledge of sexually transmitted diseases (STDs) among school-going adolescents in Europe: A systematic review of published literature. BMC Public Health.

[26] Public Health England (2016). Sex Trans Infect (STIs): Annual Data Tables.

[27] Salfa M.C., Suligoi B. (2016). Prevalence of Chlamydia trachomatis, Trichomonas vaginalis and Neisseria gonorrhoeae based on data collected by a network of clinic microbiology laboratories, in Italy. Adv. Exp. Med. Biol..

[28] ECDC (2020). Chlamydia Infection. Annual Epidemiological Report for 2018. https://www.ecdc.europa.eu/en/publications-data/chlamydia-infection-annual-epidemiological-report-2018.

[29] Rieder H.L. (1999). Epidemiologic Basis of Tuberculosis Control.

[30] Parker R. (2009). Sexuality Education in Europe: An Overview of Current Policies. Sex Educ..

[31] Drago F., Ciccarese G., Zangrillo F., Gasparini G., Cogorno L., Riva S., Javor S., Cozzani E., Broccolo F., Esposito S. (2016). Survey of Current Knowledge on Sexually Transmitted Diseases and Sexual Behaviour in Italian Adolescents. Int. J. Environ. Res. Public Health.

[32] Ciccarese G., Drago F., Herzum A., Rebora A., Cogorno L., Zangrillo F., Parodi A. (2020). Knowledge of sexually transmitted infections and risky behaviors among undergraduate students in Tirana, Albania: Comparison with Italian students. J. Prev. Med. Hyg..

[33] Bergamini M., Cucchi A., Guidi E., Stefanati A., Bonato B., Lupi S., Gregorio P. (2013). Risk perception of sexually transmitted diseases and teenage sexual behaviour: Attitudes towards in a sample of Italian adolescents. J. Prev. Med. Hyg..

[34] Orlando G., Campaniello M., Iatosti S., GRislande P.J. (2019). Impact of training conferences on high-school students’ knowledge of sexually transmitted infections (STIs). J. Prev. Med. Hyg..

[35] Napolitano F., Napolitano P., Liguori G., Angelillo I.F. (2016). Human papillomavirus infection and vaccination: Knowledge and attitudes among young males in Italy. J. Hum. Vaccines Immunother..

[36] Coronado P.J., Delgado-Miguel C., Rey-Cañas A., Herráiz M.A. (2017). Sexual and reproductive health in Spanish University Students. A comparison between medical and law students. Sex. Reprod. Health.

[37] Karamouzian M., Shahesmaeili A., Khajehkazemi R., Hooshyar S.H., Fallahi H., Haghdoost A.A., Shari H. (2017). Awareness of and knowledge about STIs among nonmedical students in Iran. Int. Perspect. Sex. Reprod. Health.

[38] Shelton J.D., Halperin D.T., Nantulya V., Potts M., Gayle H.D., Holmes K.K. (2004). Partner reduction is crucial for balanced “ABC” approach to HIV prevention. BMJ.

[39] Ahmed S., Lutalo T., Wawer M., Serwadda D., Sewankambo N.K., Nalugoda F., Makumbi F., Wabwire-Mangen F., Kiwanuka N., Kigozi G. (2001). HIV incidence and sexually transmitted disease prevalence associated with condom use: A population study in Rakai, Uganda. AIDS.

[40] Hearst N., Chen S. (2004). Condoms for AIDS Prevention in the Developing World: A Review of the Scientific Literature. Stud. Fam. Plan..

[41] Owen J., Carroll C., Cooke J., Formby E., Hayter M., Hirst J., Lloyd Jones M., Stapleton H., Stevenson M., Sutton A. (2010). School-linked sexual health services for young people (SSHYP): A survey and systematic review concerning current models, effectiveness, cost-effectiveness and research opportunities. Health Technol. Assess..

[42] European Union Agency for Sexual Services Accessing Reproductive or Sexual Health Services. https://fra.europa.eu/en/publication/2017/mapping-minimum-age-requirements/sexual-health-services.

[43] National Health Service What Services Do Sexual Health Clinics (GUM Clinics) Provide?. https://www.nhs.uk/common-health-questions/sexual-health/what-services-do-sexual-health-clinics-gum-clinics-provide/.

[44] Haldre K., Part K., Ketting E. (2012). Youth sexual health improvement in Estonia, 1990–2009: The role of sexuality education and youth-friendly services. Eur. J. Contracept. Reprod. Health Care.

